# Report of a Case of Addison’s Disease

**Published:** 1896-04

**Authors:** J. N. Hall

**Affiliations:** Denver, Colorado, Professor of Therapeutics and Clinical Medicine, University of Colorado


### REPORT OF A CASE OF ADDISON'S DISEASE.

By J. N. HALL, Denver, Colorado,

Professor of Therapeutics and Clinical Medicine, University of Colorado.

Although no autopsy could be obtained in the following case, I believe it was sufficiently typical to make it worthy of record:

J. D., female, sixteen years of age, was born in Iowa, of healthy parents, and had no family history of interest in this connection. As a baby she had a severe attack of bronchitis, and, at the age of five years, one of acute lobar pneumonia. Had also the usual children's diseases, but was pretty well generally, with these exceptions, until fourteen years of age. At that time it was noted that she was getting darker in color than normal, but it was attributed to sunburn. Her general health was not greatly affected until six months later, when the discoloration became marked. She lost some weight at this time, falling from eighty-three pounds to about seventy-three pounds. First menstruation fifteen months ago, second one year ago. Since that time has flowed nearly every month, and of late has a pale flow nearly all the time, more leucorrheal than menstrual in character.

She has had distress after meals since the beginning of the discoloration, and of late has been constipated. Appetite good, excepting during the past summer. The mother thinks the child passes an increased amount of water, and that she drinks more than normal, but she does not arise in the night for micturition. Of late she has had morning sickness.

Since September last, a period of six months, she has had marked dyspnea and palpitation upon exertion. The dyspnea is at times noticed when quiet in bed. Although she had a dry, hacking cough in the beginning, she has had none of late excepting when suffering from a cold. She formerly complained a few times of pain through the region of the heart, and once or twice in the pelvis during menstruation, but aside from these manifestations she has been free from pain. There has been no edema. She has been excessively nervous, especially at her menstrual periods. Her face has at times twitched during the night. Her knees have been sore at the menstrual epoch at times, and a fine eruption has appeared, not itching, but tender. This appeared over the hips during one menstruation. During the past summer she suffered from several attacks of hives, as she has commonly done for years.

Examination showed a slender girl, weighing but sixty-eight pounds, excessively nervous, emaciated, breasts but slightly developed. Over the face were scattered about fifty black spots, none more than one or two lines in diameter, not papular. The mother states that some of them have become smaller and perhaps may have disappeared, but of this point I do not feel at all certain. They first appeared about fourteen months ago. They are stated by Niemeyer to be characteristic of the disease.

The skin of the whole body is much darkened, and the color approaches a mahogany shade over the joints, especially those of the spine, the knees, elbows, and the right shoulder. Dr. H. B. Whitney examined her with me, and we found the lungs, kidneys, spleen, liver, pupils, and external genitals negative. No lumbar tenderness. The pulse was 120 per minute, and the heart's action very feeble. The patient was somewhat anemic and complained, even during the examination, of excessive languor.

Presenting then the typical symptoms mentioned by Addison, anemia, debility, feeble heart, irritable stomach and pigmentation, the diagnosis seemed established, as none of the other causes of similar symptoms could be found. A grave prognosis was given and a tonic treatment prescribed. She failed gradually and died in four months, the course being about two years and four months from the first symptoms.

